# Metal Hypersensitivity in Patients With Failure of Joint Prosthesis Treatment

**DOI:** 10.1155/jimr/4319686

**Published:** 2025-03-10

**Authors:** Jana Bruna, Jarmila Prochazkova, Stepan Podzimek, Lucie Himmlova, Tatjana Janatova, Alex Vinsu

**Affiliations:** ^1^Institute of Dental Medicine, First Faculty of Medicine and General University Hospital in Prague, Charles University, Prague, Czech Republic; ^2^Jessenius Faculty of Medicine, Comenius University, Martin, Slovakia

**Keywords:** arthroplasty, hypersensitivity, lymphocyte, metal, modified lymphocyte transformation test (mLTT)

## Abstract

The objective of this study is to measure lymphocyte responses to metal antigens using MELISA (memory lymphocyte immunostimulation assay) test–modified lymphocyte transformation test (mLTT) and to evaluate metal sensitization in patients with and without the need of prosthetic surgery. This study is a case-control retrospective survey. We retrospectively analyzed all patients from 2013 to 2018 who were referred to the Institute of Dental Medicine, General University Hospital in Prague, and First Faculty of Medicine, Charles University, Prague, either following joint prosthesis-related complications or as a preoperative evaluation concerning metal hypersensitivity. For the control group, we selected healthy adults from our database. A group of 127 patients aged 25–81 years was chosen, 92 of which were female and 35 were male. The patients completed a special questionnaire aimed at information regarding their health status and history of metal exposure. After clinical examination, their peripheral blood samples were taken to perform mLTT. mLTT provided quantitative lymphocyte proliferation measurement, where a stimulation index of >2 indicated metal sensitivity. For statistical analysis, the Fisher's exact test, χ^2^ test, McNemar's exact test Student's paired *t*-test were used. By comparison of the study group and control group mLTT results, it can be stated that patients of the study group showed a higher level of lymphocyte reactivity to most of the tested metal antigens (Ag [silver], Cu [copper], Fe [iron], Mo [molybdenum], Pd [palladium], Pt [platinum], Ti [titanium], and Zn [zinc]) and an elevated incidence of metal hypersensitivity to Hg (mercury), Al (aluminum), Au (gold), Co (cobalt), Cr (chromium), Ni (nickel), and Sn (tin). The evaluation of the data obtained from patients in this study confirmed a significant clinical benefit of mLTT in diagnostics of metal hypersensitivity. Our study has revealed that the patients with the need of prosthetic surgery exhibited an elevated lymphocyte response to metal antigens. This result supports a metal-specific adaptive immune response and suggests involvement of metal exposure as a trigger for their health problems. This knowledge could be helpful in effectively enhancing the treatment of patients with need of orthopedic joint prosthesis.

## 1. Introduction

Total joint arthroplasty (TJA) is the definitive treatment for severely damaged synovial joints [1] and has revolutionized the treatment of end-stage arthritic disorders [2]. TJA has excellent long-term results in terms of reducing pain and improving function and quality of life in patients with debilitating arthritic diseases [3]. The number of TJA has considerably increased over the years. Projections predict the frequency of TJAs to further increase by 300% to over 600% by the year 2030 [4]. Despite continual changes in surgical technique and implant design, the revision of total arthroplasty has not decreased over time [3]. Indications for revision TJA include septic and aseptic etiologies, whereas the causes and mechanisms of such complications often remain unidentified [1]. The large-scale recalls of metal-on-metal implants have had significant economic costs to the healthcare industry. Thus, recent studies target the identification of factors that contribute to decreasing morbidity and mortality after TJA. Besides patient's satisfaction, these factors are supposed to bring cost-effectiveness to the hospital [1, 5].

Scientific literature and clinical experience show that metals play a key role in the development of allergy [6–8]. According to case reports, allergic reactions to metal prosthetic components have been observed [9–15]. There are several retrospective [16–18] and prospective [19–21] studies evaluating metal sensitization related to joint prostheses. No evidence has shown that patients with a known metal allergy have a higher rate of failure or revision of primary TJR than those without such a history [6, 22]. Studies used patch tests and lymphocyte transformation tests (LTTs) for the evaluation of metal sensitization. The patch (epicutaneous) test is regarded as golden standard and the only test available for routine in vivo diagnosis of metal hypersensitivity. Although the test is useful in clinical praxis, it has several disadvantages [23]. MELISA (memory lymphocyte immunostimulation assay) test was developed as a modern in vitro testing LTT tool for the diagnosis of occupational drug allergy. Currently, it is scientifically proven and clinically validated blood test that detects type-IV allergy to multiple metals [24].

## 2. Patients and Methods

### 2.1. Patients

The objective of this study is to measure lymphocyte responses to metal antigens using MELISA test and to evaluate metal sensitization in patients with and without the need of prosthetic surgery.

We aimed to evaluate metal-specific adaptive immune response in patients with need of TJA. The patients were mostly referred for modified lymphocyte transformation test (mLTT) by their orthopedic surgeons, whose decision was based upon suspicion of metal hypersensitivity. For comparison, control patients without the need of prosthetic surgery were selected.

In referred patients, previous professional exposure to metal antigens was well-known or metal hypersensitivity reaction was proven, or symptoms related to the joint replacements were observed: pain, instability, swelling, or skin changes and stiffness across the periprosthetic region.

After signing the informed consent, the patients were asked to fill a special questionnaire. Possible allergy-inducing metals were identified through the questionnaire evaluation, which was then followed by laboratory testing with mLTT.

Group 1 consisted of patients with the need of prosthetic surgery (*n* = 107, 26 males, 81 females, average age 64,1 years, age range 25–81 years).

Group 1 was divided into four subgroups:

OA: patients with severe osteoarthritis (*n* = 10, 1 male, 9 female).

AA: patients with severe osteoarthritis and a positive history of metal hypersensitivity (*n* = 23, 0 male, 23 female).

1A: patients with an ongoing failure of their first arthroplasty (*n* = 43, 13 male, 30 female).

2A: patients with an ongoing failure of their at least second arthroplasty (*n* = 31, 12 male, 19 female).

OA group consisted of patients with severe osteoarthritis. Orthopedic surgeons referred these patients for mLTT testing prior to their knee or hip arthroplasty. Reasons for referral were well-known professional exposure to metals, family history of metal allergy and comorbidities.

In AA group, there were patients with a positive history of hypersensitive reactions to metals. Patients mostly experienced skin allergic reaction to metal buttons in their garments, wrist watches, and metallic jewelry. Two patients described oral allergic symptoms such as swelling and burning accompanied by dry oral cavity and feeling of metallic taste. There were two patients with polyvalent allergy. Some of the patients underwent patch skin test.

In 1A group, there were patients with failing total knee or hip endoprostheses. Patient presented with swelling, pain, aseptic loosening, and skin defects after arthroplasty. The removal of the prosthesis was considered in these patients.

Group 2A consisted of patients with repeated symptoms of implant failure. They experienced signs of inflammation, swelling, pain, itching, dermatitis, and loss of joint function in multiple metal implants or after revision surgery. The implants were already removed in 24 patients and the removal was considered in the rest of them.

Group 2 consisted of healthy controls without any prosthetic surgery (*n* = 20, 9 males, 11 females, average age 43 years, age range 25–64 years).

In the control group, there were healthy volunteers—the patients who passed preventive dental care at the Institute of Dental Medicine, General University Hospital in Prague, and First Faculty of Medicine, Charles University.

In total, 127 individuals participated in this study. In Group 1, mLTT test was performed in 74 patients with implant-related complications and in 33 preoperative patients. Out of 74 patients, the implants were already removed in 24 patients and the removal was considered in the rest of them.

This study was approved by the Ethical Commission of the General University Hospital in Prague and was performed in agreement with the Helsinki Declaration.

### 2.2. MELISA Test

After signing the informed consent, the patients were asked to fill a special questionnaire. This questionnaire was focused on exposure to metals with questions regarding family history, exposure to metals in the past and present, allergy occurrence, and smoking experience.

Some of the items closely related to the concept of the study are shown in Table 1.

Possible allergy-inducing metals were identified through the questionnaire, which was then followed by laboratory testing with MELISA test. The MELISA test is based on evaluating the proliferation of peripheral blood memory cells in vitro after incubation with metal salts [25, 26].

For the purpose of our study, we followed the methods of Podzimek et al. [27]. Ten milliliters of peripheral venous blood were collected and centrifuged to provide the patients' autologous serum. Heat-inactivated autologous serum was used for the cultivation of lymphocytes. Thirty milliliters of peripheral venous blood was collected and mixed with an equal amount of RPMI 1640 (Roswell Park Memorial Institute) medium containing 10 mM HEPES (4-(2-hydroxyethyl)-1-piperazineethanesulfonic acid), gentamycin, and glutamine. The blood was layered on a Ficoll-Paque gradient (Histopaque, Sigma–Aldrich) and centrifuged at 600 *g* for 30 min. Mononuclear cells were collected from the interface, washed twice, and then mixed with 5 ml of RPMI 1640 medium containing 20% of inactivated autologous serum. Plastic-adherent cells were partially depleted from leukocyte suspension by incubation on plastic surfaces for 40 min at 37°C. After incubation, the lymphocytes were counted and diluted with RPMI 1640 enriched with 10% autologous serum and glutamine into a final dilution of 1 × 10^6^ cells/ml. Lymphocytes were cultivated for 5 days with metal salt solutions in an atmosphere of 5% CO_2_ in humidified air at 37°C. All patients in this study were tested for nickel (Ni), chromium (Cr), Fe, titanium (Ti) (in the form of chloride and oxide), aluminum (Al), molybdenum (Mo), Cu, platinum (Pt), cobalt (Co), and zirconium [27]. Details of the specific metal salts and their concentrations are described by Stejskal et al. [25]. Control cultures were incubated under the same conditions in the absence of metal salt solutions. As a positive control, lymphocytes were cultivated with Pokeweed mitogen (10 µg/ml, Sigma, USA). After 5 days' cultivation, lymphocyte cultures were split into two parts [27]. One part was used to measure lymphocyte proliferation by ^3^H thymidine incorporation (Perkin Elmer, USA), as described in the article by Stejskal et al. [25]. The second part was frozen at −20°C to determine proinflammatory cytokine production. The rate of lymphocyte proliferation in metal-treated cultures was compared to the rate in nonstimulated cultures and evaluated by a stimulation index (SI): counts per minute in metal-treated cultures divided by counts per minute in nontreated cultures. An SI of less than 2 was regarded as a negative reaction, SI 2.01–5 as a positive reaction and SI higher than 5 was regarded as a strongly positive reaction [27].

### 2.3. Statistical Analysis

This study is a case-control retrospective survey.

Comparisons of categorical variables among groups were performed with the *χ*^2^ test or Fisher's exact test. For comparisons of continuous variables, the Student‘s paired *t*-test and McNemar's exact test were used. The statistical significance level was set at *p*  < 0.05. All measured data were normally distributed.

## 3. Results

We analyzed a total of 127 patients.

Two basic groups of subjects were defined: Group 1: study group and Group 2: control group.

The results of hypersensitivity testing were evaluated by comparing results between the two basic groups. In addition, we compared the incidence of metal hypersensitivity between the subgroups of the study group.

**Figure 1:**
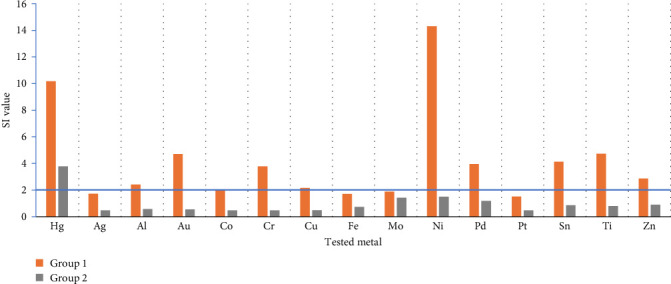
The mean values of stimulation index for each tested metal in basic groups.

The mean values of the SI for each tested metal in basic groups are shown in Figure 1. The orange columns belong to the results of MELISA tests in Group 1 and the gray columns represent the results of MELISA test in Group 2. From this figure, it is obvious that the mean value of SI for each tested metal in Group 1 is higher than in Group 2, with a significant difference (*p*  < 0.05) found in all metals except Mo.

The highest values of SI in Group 1 were obtained for Ni followed by mercury (Hg), (strongly positive reaction). Positive reaction was found to Ti, gold (Au), tin (Sn), palladium (Pd), Cr, zinc (Zn), Al, copper (Cu), and Co. Negative reaction was indicated by SI value below 2 for Mo, silver (Ag), iron (Fe), and Pt. The values of SI for Ni and Hg are distinctly higher than for other metals. In the control group, the only positive mean value of SI was obtained for Hg.

**Figure 2:**
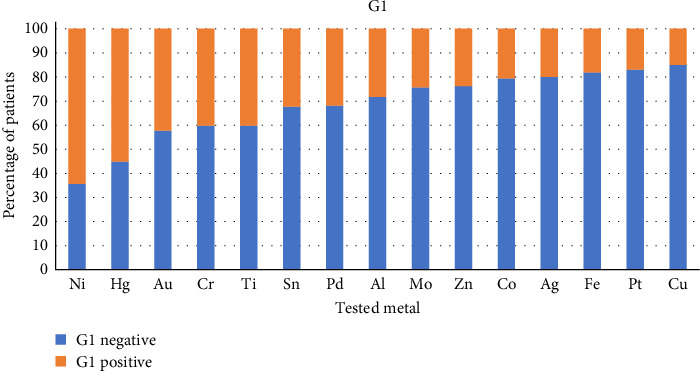
The percentage of patients with positive and negative reactions for each tested metal in Group 1.

**Figure 3:**
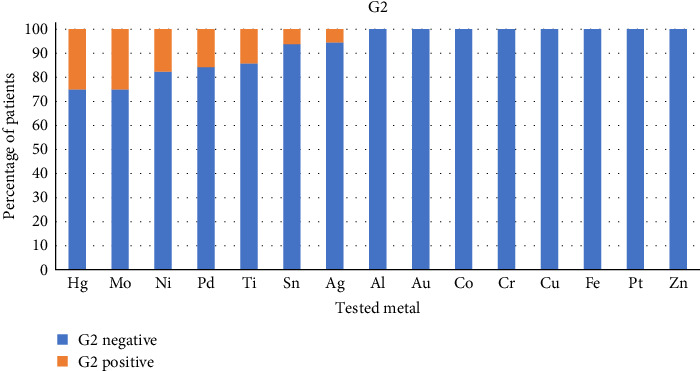
The percentage of patients with positive and negative reactions for each tested metal in Group 2.

Figures 2 and 3 show the percentage of patients with positive and negative reactions for each tested metal in Group 1 (Figure 2) and in Group 2 (Figure 3).

In each column, the orange part represents the percentage of positive reactions, and the blue part represents the percentage of negative reactions to the tested metal.

The statistical analysis has revealed the difference between Groups 1 and 2 in lymphocyte reactivity to some of the tested metals. In Group 1, we have found a higher proportion of positive lymphocyte reaction to Hg, Al, Au, Co, Cr, Ni, and Sn than in Group 2.

Further, we separately compared reactivity to metal antigens between four subgroups. For illustration, we present Tables 2–4. They show the number and percentage of patients with positive and negative reactions to Hg, Co, and Ni in all study subgroups.

Comparison of Table 2 vs.  Table 3 showed us that the incidence of reactivity to Hg is greater than to Co for most of the study subgroups. For this purpose, we used McNemar's exact test and the statistical significance level was set at *p*  < 0.05. Value smaller than 0.05 was found in 1A, 2A, and AA. Thus, it can be stated that there is greater incidence of reactivity to Hg than to Co in patients with severe osteoarthritis and a positive history of metal hypersensitivity and patients with an ongoing failure of arthroplasty. Results are shown in Table 5.

Similarly, we compared Table 2 and Table 4. No value smaller than 0.05 was found. Thus, it cannot be stated that there is a statistically significant difference between incidence of reactivity to Hg and Ni among subgroups. Results are shown in Table 6.

In addition, we compared reactivity to metal antigens between four study subgroups. Results are shown in Table 7. By comparing mLTT test results between four study subgroups, it cannot be stated that there is a significant difference between incidence of metal hypersensitivity to any of the tested metals.

Overall, it can be stated that patients of Group 1 showed an increased (SI higher than 2) level of lymphocyte reactivity to the most of tested metal antigens (Hg, Ag, Al, Au, Co, Cr, Cu, Fe, Mo, Ni, Pd, Pt, Sn, Ti, and Zn) and a higher proportion of increased lymphocyte reactivity to Hg, Al, Au, Co, Cr, Ni, and Sn than Group 2, which may correspond with an elevated incidence of metal hypersensitivity in the study group.

Comparison of lymphocyte reactivity between the subgroups revealed no statistically significant differences. However, there is an elevated incidence of metal allergy in all subgroups: AA, OA, 1A, and 2A in comparison to the control Group 2.

Our study has revealed that the incidence of reactivity to Hg is greater than to Co for most of the study subgroups (1A, 2A, and AA). This paired difference is highly statistically significant.

## 4. Discussion

Implantation of biomaterials and medical devices is followed by the sequence of local events leading to progressive integration of the implant within bone and the surrounding musculoskeletal tissues [2, 28]. The eventual outcome of the implant insertion is dependent on the characteristics of the implant, the precision of the surgical technique and operative environment, and the biological milieu of the host [2]. Biocompatibility is an essential requirement of a biomaterial. A biocompatible material disrupts the normal body function as little as possible [1]. In practice, no synthetic material is completely harmonious with the living environment [29]. Metals are used for orthopedic implants because of their mechanical properties, such as weight-to-strength ratio and good biological performance. However, metallic devices are prone to wear and corrosion, particularly in aqueous environments under extreme conditions [1, 30]. Corrosion is defined as a spontaneous and progressive loss of material caused by the surrounding environment [30]. All metals in contact with biologic systems undergo some degree of corrosion [6, 29]. Wear is the damaging, gradual removal, or deformation of material at solid surfaces [31]. The generation of byproducts from joint replacements is inevitable, due to repetitive loading of the implants [32]. Both wear and corrosion may increase the total surface of the metallic biomaterial and consequently the concentration of metal ions in the human body. The metallic debris produced after implantation may contribute to a hypersensitive reaction because metal ions released from total joint prosthesis components form in the biologic conditions haptens and tend to form hapten–carrier complexes [33]. Such complexes can be taken up by Langerhans cells and recognized by T-lymphocytes as antigens, which might trigger a specific immune response [34]. Hallab et al. [35] observed the involvement of a specific lymphocyte subtype (Th1) in the metal reactivity response to total joint replacements. This Th1 subpopulation of T-cells is associated with delayed-type hypersensitivity (DTH). Antigen-specific T_DTH_ cells are characterized by their cytokine secretion profile [7]. Activated lymphocytes release powerful proinflammatory cytokines such as interferon-*γ* (IFN-*γ*), interleukin-1 (IL-1), and IL-2, which can promote activation of macrophages (secretion of tumor necrosis factor-*α* [TNF-*α*]) and osteoclast activity and inhibit osteoblast activity. Given that lymphocytes are present around implants, it is likely that metal-induced lymphocyte reactivity may contribute to the cascade of events leading to osteolysis and aseptic loosening [7, 29]. The effect of metals on cytokine production has already been proven in the past. The production of selected proinflammatory cytokines in patients with failed orthopedic implants was measured by Podzimek et. al. [27] and Christiansen et al. [36]. It is not the subject of our study, but it can be one of the directions for future investigation.

As mentioned above, implant-related hypersensitivity reactions are generally type-IV reactions, delayed cell-mediated immune responses [7], and can occur either in the postoperative period or months and even years later [37]. Testing for metal hypersensitivity is conducted in vivo using skin patch testing. However, skin reactions are different compared to deep tissue layers and joint environment [38, 39]. Thus, patch testing is not reliable for metal hypersensitivity testing prior or after arthroplasty and its results might not reflect the real immunological response to the metal concerned [7]. In vitro lymphocyte proliferation testing (also known as lymphocyte transformation testing, or LTT) represents more clinically appropriate method of assessing peripheral and peri-implant lymphocyte reactivity to metals [7, 40], because it involves measuring the proliferative response of lymphocytes following activation. In our study, we used MELISA test to provide T lymphocyte proliferation measurement, where a SI of >2 indicated metal hypersensitivity.

Study subjects (Group 1) demonstrated higher levels of lymphocyte proliferation when compared to healthy controls. The mean values of SI were significantly elevated in study group (Group 1) for all tested metals except Mo. We have also observed elevated incidence of metal sensitivity to Hg, Al, Au, Co, Cr, Ni and Sn in the study group. Therefore, our hypothesis that lymphocytes from subjects with the need of prosthetic surgery and positive history of metal-related health issues would demonstrate a nonspecific hyperresponsiveness to implant alloy metals was supported by our results. In addition, we compared the lymphocyte reactivity between the subgroups of Group 1. However, we have not found any statistically significant differences in lymphocyte reactivity between the subgroups of patients with severe osteoarthritis, patients with severe osteoarthritis and a positive history of metal hypersensitivity, patients with an ongoing failure of their first arthroplasty or their at least second arthroplasty. Therefore, we cannot state that patients who have had an allergic reaction to a metallic device or to jewellery are more likely to have increased lymphocyte reactivity than those with no such history.

Further, we want to discuss some of the tested metals and their antigenic potential separately and in more detail. In the patients of Groups 1 and 2, the mean value of SI over 2 was obtained only for Hg. This finding corresponds with common knowledge that Hg is well-known allergen. Hg affects the body mainly in two ways, through toxic and immunologic reactions. Studies indicate that there is no safe limit of Hg in humans. Even with the same amount of exposure, there is great variation in both the level stored in the body and the effects of the exposure [41]. Despite its toxicity, the use of Hg and Hg compounds has been widespread in medicine in the past. Nowadays, the preservative thiomersal is added to some adult vaccines [42]. Without a preservative, multidose liquid presentations of vaccine are vulnerable to bacteriological contamination that can result in death or serious illness of the recipient. In wealthy countries, monodose, thiomersal-free vaccines have been introduced as a precautionary measure in almost all childhood vaccines [43].

In general, humans are exposed to Hg mostly from dental amalgam filling and fish consumption [44–47]. The immunological effects of Hg are hypersensitivity or autoimmunity. This metal has been proven to be a risk factor for the development of various autoimmune diseases such as autoimmune thyroiditis, multiple sclerosis, and unspecific symptoms such as chronic fatigue and fibromyalgia [41]. Hg has a strong allergic potential [26] as demonstrated by the results of our study.

In Group 1, most of our patients were tested positive for Ni (64%). Ni hypersensitivity is very common in the general population. Approximately, 15% of the population have been reported as being hypersensitive to Ni [48]. Ni is a transition metal with an autoimmune potential. His potential risk resides in the physic-chemical properties; Ni has a strong affinity to sulfur groups present in two amino acids; methionine and cystine. Consequently, they form a strong bond with enzymes and other proteins in the body, altering their structure and rendering them foreign to the immune system and susceptible to an autoimmune attack [41, 42]. In our study, 17% of the healthy controls demonstrated sensitivity to this metal.

The Ni allergy usually occurs in association with a cross-reactivity to Co [49, 50]. In Group 1, 20% of our patients were tested positive for Co and it was nearly always (87.5%) associated with a positive reaction to Ni.

Co is the second most common metal allergen. Patients tested with the European baseline series between 2015 and 2018 showed a prevalence of positive tested individuals of 5.4%. Metal implants made of Co alloys have been found to release high concentrations of Co^2+^ ions into the blood. Co acts as a cofactor for several enzymes in humans and other organisms. Exposure to Co may lead to metal allergy, chronic and acute respiratory diseases, metallosis, and increased risk of cancer. Therefore, its content has recently been regulated by EU legislation including a temporary generic concentration limit of ≥0.1% [51].

Based on comparison of Table 2 vs.  Table 3, it is obvious that the incidence of reactivity to Hg is greater than to Co for most of the study subgroups. This paired difference is highly statistically significant and a very important finding of this study since Hg reactivity is known to be higher than Co but there is no published data supporting such LTT results in patients with a failing joint replacement. According to Wawrzynksi et al. [52], most orthopedists and dermatologists agree that an alternative prosthesis should only be considered for patients with a history of allergy to a metal in the standard implant [52]. Podzimek et al. [27] revealed that hypersensitivity to failed implant composition was determined in 40% of patients with failed implants. Since joint replacements do not contain Hg, our study rather supports the opinion that it is difficult to determine whether sensitization is a cause or a consequence of implant failure [53].

High level of hypersensitivity was also found in reaction to Ti. Forty percentage of our study patients were tested positive for Ti. Nowadays, the hypersensitivity to Ti is recorded more frequently. People are exposed to Ti (primarily titanium dioxide) from implantable medical devices and other sources such as sunscreens, toothpastes, and food [54, 55]. Ti alloys currently belong to materials of choice for biological applications [56, 57]. However, under biological circumstances of the human body in combination with cyclic loads, Ti compounds can undergo corrosion [58]. Ti orthopedic implants form a passivating superficial TiO_2_ film that can prevent extensive corrosion. However, implant wear degrades TiO_2_ layer, resulting in corrosion [1]. Contrary to common belief, Ti is not inert and can induce clinically relevant hypersensitivity reactions as well as other immune dysfunctions [59, 60]. The values of SI obtained in our study have confirmed increased hypersensitivity to Ti.

Authors have not measured reactivity to zirconium, which is a part of zirconium ceramics. Ceramic materials may be a suitable alternative to metallic materials because they do not suffer from corrosion and wear in biological environments [12, 61, 62]. Further evaluation using mLTT should be performed to hopefully provide the essential knowledge of metal hypersensitivity responses to all basic implant metals.

We aimed to relate our results to the existing research. Hallab et al. [63] observed elevated lymphocyte response to Ni and Co in patients with osteoarthritis and a history of metal sensitivity when compared to other patients with osteoarthritis and no previous allergy and patients after hip arthroplasty. According to our results, there was no significant difference in reactivity to Cr between the study subgroups. Niki et al. [64] identified a significant association between the presence of Cr-sensitivity and development of eczema after total knee arthroplasty (TKA) and suggested that surgeons should undertake routine preoperative screening for metal sensitivity, particularly to Cr. Our findings are in accordance with these results. However, our study group includes different types of arthroplasties; total hip arthroplasties (THAs) as well as TKAs. This heterogeneous design of the study population does not allow for further comparison with previous studies based exclusively on THA or TKA populations. Therefore, we suggest distinguishing between TKA and THA in future studies in order to provide better comparability. We also suggest including a control group comprising orthopedic patients without implant-related complications for comparison in future research. We believe that comparison of implant performance in prospective groups with and without metal reactivity can help to correlate implant performance with lymphocyte reactivity.

Limitations to our study are notable including the retrospective design and the small sample size of control patients. When comparing incidence of metal sensitivity between Groups 1 and 2, a small number of patients were studied (especially patients sensitive to Zn and Mo). Both referred patients and control patients came from the Czech Republic and most of them lived near Prague. Thus, medical data were collected from a specific population in a particular geographic area and cannot perfectly represent the country in general.

Extraneous variables such as age may account for the differences in lymphocyte response to metal challenge [63]. In our study, the patients with the need of prosthetic surgery were elder (average age 64.1 years) than those in the control group (average age 43 years). This noticeable age disparity between the groups can account for the variations in the stimulations index, rather than metal exposure. We need to address this potential confounding factor.

Further, gender distribution of the study group differs from the control group. While the control group shows an almost equal number of male and female patients, the study group is comprised predominantly of women (75.7%). The incidence of metal sensitivity appears to be higher in elderly women [65]. Gender also seems to play a role in self-reporting of metal hypersensitivity. Nam et al. [66] retrospectively investigated 1495 patients with total knee or hip arthroplasty regarding the presence of a metal allergy. The incidence of patient-reported metal allergy was 4% and 98% of them were female [66].

Peña et al. [67] studied the psychologic consequences of metal hypersensitivity in patients who self-reported metal allergy and reported worse clinical results in patients who received hypoallergenic implants than in those receiving conventional implants. Their results seem to be related not to the implant itself but to the patients' humoral state in response to the allergy and the psychological distress they experienced [67]. In general, it has been proven that psychological distress or psychiatric pathology negatively influences clinical outcomes after joint arthroplasty [68–71]. However, whether metal hypersensitivity impacts patients' psychologic factors or a true immune response to metal implants remains unclear [72]. Metal sensitivity may exist as an extreme complication in only a few highly susceptible patients (i.e., less than 1% of joint-replacement recipients), or it may be a more common subtle contributor to implant failures [7]. We believe that metal hypersensitivity in patients with failing arthroplasty is a complex topic and will attract broad attention in the future because the use of metallic implants and the expectations of implant durability and performance are increasing.

## 5. Conclusions

The number of TJA patients worldwide rises and demand for revision TJA occurs. Poor surgical technique and implant design cause implant failure, yet 10%–15% of patients experience aseptic implant failure despite well-designed implants and meticulous surgical technique [1].

Patients with metal hypersensitivity may suffer from numerous symptoms [73–77] associated with an activated immune system. The experience of our team from recent years has shown growing interest in orthopedists in mLTT due to an increased number of aseptic implant failures. Our work shows that conditions with pathological immune reactivity to metals occur in patients with well-known professional or environmental exposure to metals or a positive history of metal hypersensitivity who are about to receive a joint replacement. We have also observed this immune activity in patients with a failing arthroplasty. Patients from abovementioned groups exhibited an elevated lymphocyte response to most of the metals used in orthopedic implants or osteosynthesis devices.

However, it is still unclear whether metal hypersensitivity causes implant failure or vice versa. It is likely that some combination of these phenomena occurs whereby implant-loosening promotes immunogenic reactions, which in turn act to potentiate the loosening cascade [7].

When patients with hypersensitivity to metals present for surgery where metallic prostheses are required, problems arise about the choice of the prosthesis.

Hypoallergenic implants were shown as viable alternatives for patients with self-reported or confirmed metal hypersensitivity. However, concerns remain over their long-term outcomes [78, 79].

It has been proven that psychological distress or psychiatric pathology negatively influences clinical outcomes after joint arthroplasty [67, 68]. We believe that this aspect is fundamental in the presurgical evaluation of patients.

Ultimately clinical judgment should be used to determine which patients may benefit from hypoallergenic implants.

Testing for implant-related hypersensitivity has been historically conducted in vivo by skin testing. However, there is continuing concern about the applicability of patch testing to the study of immune responses to implants [7].

In vitro DTH testing remains a labor intensive and clinically unpopular means of assessing metal hypersensitivity.

However, based on the mLTT report, the implant material to be used in a patient allergic to a particular metal may be chosen to avoid the possible allergy reaction. If the commonly used prostheses cannot be used, the prosthesis can be specially procured for the patient [80].

According to the obtained results, routine in vitro hypersensitivity testing before total joint replacement in patients with a history of health problems associated with metal exposure should be considered.

Authors believe that it is necessary to perform multiple tests on individual patients to reveal metal hypersensitivity directly related to joint replacements. In further research, we suggest performing mLTT test before implantation; during the service of the device; and, in the case of an adverse outcome, before and after removal of the device.

## Figures and Tables

**Table 1:** The main items of the questionnaire.

Presence of metallic implantable devices	Yes/no, what type and material, how long
Implant-related problems	Yes/no, specification and duration
Metal allergy	Yes/no, specification of tests
Reaction after contact with metal items (jewelry, watches, and piercing)	Yes/no, burning, itching, swelling, rash, and type of material
Smoking experience	Yes/no, how long and how much
Professional exposure to metals	Yes/no, what metal and how long
Environmental exposure to metals	Yes/no, what metal and how long

**Table 2:** The number and percentage of patients with positive and negative reactions to Hg in subgroups OA, AA, 1A, and 2A.

Hg	Negative	Positive	Total amount	Negative (%)	Positive (%)
1A	16	21	37	43.2	56.8
2A	14	16	30	46.7	53.3
AA	9	12	21	42.9	57.1
OA	4	4	8	50.0	50.0
In total	43	53	96	44.8	55.2

**Table 3:** The number and percentage of patients with positive and negative reactions to Co in subgroups OA, AA, 1A, and 2A.

Co	Negative	Positive	Total amount	Negative (%)	Positive (%)
1A	35	8	43	81.4	18.6
2A	21	10	31	67.7	32.3
AA	19	4	23	82.6	17.4
OA	8	2	10	80.0	20.0
In total	83	24	107	77.6	22.4

**Table 4:** The number and percentage of patients with positive and negative reactions to Ni in subgroups OA, AA, 1A, and 2A.

Ni	Negative	Positive	Total amount	Negative (%)	Positive (%)
1A	14	27	41	34.1	65.9
2A	10	21	31	32.3	67.7
AA	7	15	22	31.8	68.2
OA	5	5	10	50.0	50.0
In total	36	68	104	34.6	65.4

**Table 5:** Results of McNemar's exact test used for comparison between Tables 2 and 3.

Subgroup	*p*-value
1A	0.006
2A	0.121
AA	0.008
OA	Small sample size

**Table 6:** Results of McNemar's exact test used for comparison between Tables 2 and 4.

Subgroup	*p*-value
1A	0.343
2A	0.387
AA	0.752
OA	Small sample size

**Table 7:** Results of statistical analysis—comparison of positive reactivity to metal antigens among four study subgroups.

Metal	Hg	Ag	Al	Au	Co	Cr	Cu	Fe
*p*	0.978	0.358	0.627	0.125	0.484	0.123	0.949	0.458

**Metal**	**Mo**	**Ni**	**Pd**	**Pt**	**Sn**	**Ti**	**Zn**	

*p*	0.450	0.753	0.234	0.955	0.697	0.510	0.355	

*Note:* Comparisons of variables were performed with the *χ*^2^ test for contingency tables. The statistical significance level was set at *p*  < 0.05. No value smaller than 0.05, that is, there was no significant difference in reactivity to metals between subgroups AA, OA, 1A, and 2A.

## Data Availability

The data presented in this study are available upon request from the corresponding author.

## Nomenclature

mLTT: Modified lymphocyte transformation test
TJA: Total joint arthroplasty
TKA: Total knee arthroplasty
THA: Total hip arthroplasty
SI: Stimulation index
RPMI: Roswell Park Memorial Institute
HEPES: 4-(2-hydroxyethyl)-1-piperazineethanesulfonic acid
MELISA: Memory lymphocyte immunostimulation assay
DTH: Delayed type hypersensitivity.
